# Unique Challenges in Biomarkers for Psychotic Disorders

**DOI:** 10.3390/brainsci14010106

**Published:** 2024-01-22

**Authors:** Eric Y. H. Chen, Stephanie M. Y. Wong

**Affiliations:** 1 Department of Psychiatry, School of Clinical Medicine, LKS Faculty of Medicine, The University of Hong Kong, Hong Kong; 2 Department of Social Work and Administration, The University of Hong Kong, Hong Kong; steph.my.wong@gmail.com

## 1. Background

Biomarkers are observations that provide information about the risk of certain conditions (predictive) or their underlying mechanisms (explanatory) [1,2]. In the field of psychotic disorders, biomarker research has been gaining momentum for several reasons. First, psychotic disorders are diagnosed based on experiential symptoms; oftentimes, there is a preference for non-experiential signals in medicine, as they are considered more repeatable and therefore more objective. Second, experiential information is considered the final phenomenon in a complex cascade of pathophysiological mechanisms, the processes of which are organised in a hierarchy of levels, ranging from genetics, protein, cellular, and brain network to cognitive, functional, and experiential. Third, biomarkers are widely supposed to be located closer to the molecular end of the spectrum and may thus “underlie” and “provide explanatory accounts” for higher-level phenomena (such as experiential contents); the assumption that biomarkers are located more proximal to molecular mechanisms may help reduce the complexity in the cascading hierarchical system of pathophysiological mechanisms [1].

## 2. Recent Search for Psychosis Biomarkers

Searches for optimal biomarkers in psychotic disorders, such as schizophrenia, have been challenging and have so far been rewarded with only limited success [3,4]. There are, nevertheless, several considerations required. For instance, the brain system involved in psychotic disorders (“associative” systems in the brain) is largely complex and involves a large number of interconnected elements [5,6]. Key pathophysiological events likely reside in the emergent behaviour of networks of interacting elements at different hierarchical pathophysiological levels, rather than in any individual component. Importantly, in such highly connected systems, it can be challenging to demarcate what exactly constitutes the individual elements and what constitutes collective emergent behaviours. The relative inaccessibility of brain processes further makes technical access to detailed information challenging. In addition, there are also significant knowledge gaps between each of these five major levels of analysis: (1) from molecular to cellular; (2) from cellular to neural networks; (3) from neural networks to brain systems; (4) from brain systems to neurocognitive functions; and (5) from neurocognitive functions to experiential phenomena. Empirical mapping between many of these levels remains limited to date [7,8].

Despite these difficulties, many attempts have been made to complete this jigsaw puzzle. For example, recent initiatives include the endophenotype approach and the Research Domain Criteria (RDoC) initiative [1,2,9]. The endophenotype approach attends to potential genetic markers on the pathophysiological hierarchy and operationalises the criteria required for an endophenotype. The RDoC framework focuses on well-defined neurocognitive markers and attempts to demarcate the “units” of “elements” at the pathophysiological level. Both initiatives have advanced the field’s work in identifying some local pieces of the jigsaw puzzle. In some cases, further areas of complexity have been uncovered, as reflected in the increasing interests in markers for inflammation or oxidative stress in schizophrenia [10,11]. However, biomarkers that are decisive for clinical prediction are yet to be identified [2,4].

## 3. Challenges in Biomarker Identification

One of the key paradoxes in psychotic disorder biomarkers is the observation that despite the anticipation of biomarkers being more objective indicators of risk and experiential in nature, symptoms still surpass biomarkers in terms of predictive power [12,13]. One of the most extensively studied clinical areas is the prediction of psychotic disorder onset from the clinical high-risk state [14,15]. The predictive power of biomarkers (such as neuroimaging, cognitive, or molecular markers) in addition to symptoms has so far proved relatively low [16]. This empirical observation should compel us to reconsider our position on experiential signals as biomarkers. Are there any blind spots in the great pathophysiological jigsaw puzzle? What information can be obtained at the experiential level, which may render them more reliable markers rather than being discarded [17]? Indeed, more recently, symptom networks have been proposed as self-sustaining systems in which symptoms can self-regulate their emergent behaviour, highlighting the relevance of not only molecular but also experiential-level signals [18,19,20]. How more information can be distilled from experiential “markers” should be given more consideration [13,21].

Some important missing bridges in the biomarker hierarchy for psychotic disorders include those that are associated with their core, defining symptoms: hallucinations and delusions. Most of the existing neurocognitive measures studied do not directly address psychotic symptoms, but rather target neurocognitive deficits (i.e., in attempt to fill in the jigsaw pieces located some distance away from the core missing pieces) [22]. This indirect targeting of deficit phenomena (failure of performance) to try to explain spurious functions (hallucination and delusions) is justified by the Jacksonian—or more accurately, Reynoldian—suggestion that brain functions consist of hierarchical layers of function with inhibitory interactions, such that the loss of functioning (deficit) at a “higher layer” may result in disinhibition and spurious expression of anomalous function at a “lower layer” [23]. Of course, this requires the assumption of a close topographic mapping between the two layers, for which there is still little evidence. This deficit-focused approach is likely an inadvertent consequence of the use of a neuropsychological paradigm in psychosis cognition research, which starts with a known brain lesion and tries to identify the cognitive functions that are affected. This information is then used in reverse in other clinical conditions to infer the site of brain lesions from the observed neurocognitive deficits. Such a paradigm has worked well in neurological scenarios involving the more peripheral sensory and motor brain functions, where one-to-one structure-to-function mapping is more evident. Nevertheless, it may not work as well in a more central associative brain system where connections are typically in many-to-many connected networks [22,24,25]. After all, the deficit-based neuropsychological approach is appealing because of the availability of standardised testing material which depends on neutral stimuli with little individual meaning and which, as a result, is considered to be less vulnerable to “experiential contamination”, although it is important to note that the use of neurocognitive functions to identify brain dysfunction in psychosis has met with success mostly via mapping to functioning loss rather than to psychotic symptoms [26]. 

## 4. Example of Relevant Proximal Biomarker

A notable recent success in the identification of psychosis markers is the exploration of the roles of dopamine (brain level), salience (cognitive level), and delusions (experiential level) [27,28]. The increasing efforts in molecular imaging investigation have led to the identification of increased presynaptic dopamine synthesis as one of the key pathophysiological processes in psychosis represented a significant step towards completing the said jigsaw puzzle [29]. The fact that dopamine synthesis is related to the experience of delusion suggests its core role in psychotic disorders and provides a background in which the action of antipsychotic medication in the downstream post-synaptic dopamine receptors could be appraised. However, the same approach also discovered that non-dopamine-related processes are involved in the treatment-non-response psychotic phenomena [30,31].

This significant progress has implications for biomarker search. First, it highlights the need for technically accessible biomarkers more proximal to dopamine synthesis capacity. The use of molecular positron emission tomography imaging to assess dopamine synthesis is important but currently prohibitive for larger-scale implementation in terms of cost and patient burden (e.g., up to 90 min in a scanner) [32,33]. More portable alternative biomarkers could be informative, particularly if they could be repeated in real-life ecological settings. One of the potential candidates is spontaneous blink rate, which has been linked to central dopaminergic activities [34,35,36]. Advances in technology have also enabled the use of more accessible tools for capturing real-time biological and cognitive markers for managing symptoms, detecting possible cases of relapse, and guiding treatment options (e.g., remote smartphone-based assessments of self-rated symptoms and facial and vocal markers, in-the-moment ecological momentary assessments and interventions, passive GPS mobility data, and actigraphy and smartwatches) [37,38,39,40]. How such technological tools may be utilised to facilitate more accessible identification of biomarkers in psychosis should warrant further investigation. 

## 5. Criteria for Optimal Biomarkers

So far, some deficit biomarkers mapping to performance deficits in psychotic disorders have been identified, with few prominent markers for the experiential dimension of psychosis. As the clinical course of psychotic disorders is observed to fall into several related but not entirely dependent dimensions, it is suggested that optimal biomarkers should possess the following characteristics: (1) are proximal to key pathophysiological processes; (2) can be mapped to well-established positions within the known pathophysiological cascade (with well-defined links to higher- and lower-level components in the hierarchy); (3) are discrete with well-defined boundaries; (4) are portable, low-burden, and can be reliably assessed; (5) are feasible to be monitored through momentary ecological methods; (6) would not be interfered with by treatment processes; and (7) have predictive capacity for the clinical course of the disorder. It is acknowledged that fulfilling all the above criteria would be extremely challenging; some compromises may thus have to be made by clinical researchers to optimise the use of selected batteries of complementary markers with good characterisation of their functions. Such an effort would nevertheless undoubtedly facilitate advances in knowledge of the nature of psychotic disorders and inform more optimal preventative intervention and treatment possibilities. 
